# Role of YY1 in the pathogenesis of prostate cancer and correlation with bioinformatic data sets of gene expression

**DOI:** 10.18632/genesandcancer.12

**Published:** 2014-03

**Authors:** Vaishali Kashyap, Benjamin Bonavida

**Affiliations:** ^1^ Department of Microbiology, Immunology, and Molecular Genetics, David Geffen School of Medicine, Jonsson Comprehensive Cancer Center, University of California at Los Angeles, Los Angeles, California

**Keywords:** Bioinformatics, Prognosis, Prostate Cancer, Resistance, Yin Yang 1

## Abstract

Current treatments of various cancers include chemotherapy, radiation, surgery, immunotherapy, and combinations. However, there is a need to develop novel diagnostic and therapeutic treatments for unresponsive patients. These may be achieved by the identification of novel diagnostic and prognostic biomarkers which will help in the stratification of patients' initial responses to particular treatments and circumvent resistance, relapses, metastasis, and death. We have been investigating human prostate cancer as a model tumor. We have identified Yin Yang 1 (YY1), a dysregulated transcription factor, whose overexpression correlated with tumor progression as well as in the regulation of drug resistance and the development of EMT. YY1 expression is upregulated in human prostate cancer cell lines and tissues. We postulated that YY1 may be a potential biomarker in prostate cancer for patients' stratification as well as a novel target for therapeutic intervention. We used Bioinformatic gene RNA array datasets for the expression of YY1 in prostate tumor tissues as compared to normal tissues. Interestingly, variations on the expression levels of YY1 mRNA in prostate cancer were reported by different investigators. This mini review summarizes the current reported studies and Bioinformatic analyses on the role of YY1 in the pathogenesis of prostate cancer.

## INTRODUCTION

Prostate cancer is a cancer that forms in the tissues of the prostate gland, a tubulo-alveolar exocrine gland that surrounds the male bladder and urethra and is part of the male reproductive system. The primary function of the prostate in the body is to secrete a milky alkaline fluid that is a major constituent of semen. Other non-peptides and prostate-specific antigens are also produced by the prostate and are secreted out. The basicity of secreted fluid helps neutralize the acidity of the vaginal tract and allows for longer survival, better motility, and protection for the sperm [1].

Cancer in the prostate gland occurs usually in men of age 65 or older and once formed, may metastasize from the gland to other parts of the body. Ethnicity and a high fat diet are also very significant risk factors. While morbidity rates have increased significantly, almost two fold from the year 1990 to 2007, there have also been important developments in diagnosing prostate cancer with the help of recent technologies[1] [2]. Symptoms of prostate cancer include a slow and bloody urinary stream and pain in the pelvic bones. When and after urinating, the urine may leak and the patient may also have trouble emptying out all of the urine. Cancer in the prostate gland may also cause difficulty during sexual intercourse and the patient may experience erectile dysfunction. Erectile dysfunction can also increase when the patient goes through surgery or radical prostatectomy; this is primarily due to the injury done on nerves caused by inflammatory responses and thermal injury [1].

A physical test, a CT scan, a biopsy, or circulating levels of Prostate-Specific Antigen test (PSA Test) are needed to determine whether prostate cancer is present. For a prostate biopsy, a tissue sample is taken from the prostate gland and is examined under a microscope and scanned for cancerous cells with the help of a transrectal ultrasound (TRUS). The TRUS allows for higher resolution probes and, consequently, facilitates visualization of tissue samples [3]. The more differentiated the cancerous cells are from the normal healthy cells, the more severe the cancer is. This test may not always be accurate because one may miss the cancerous tumor tissue in the examined field. Another procedure to check for prostate cancer is the PSA test. PSA is a glycoprotein produced by the prostate gland and the PSA test measures the blood level of the PSA protein. High levels of PSA indicate a high probability of prostate cancer because PSA production increases in men with prostatic diseases possibly due to the structural distortion of the gland itself [4]. However, the PSA test is not fully diagnostic as high levels of PSA may also result from benign prostatic hyperplasia, or BPH, which occurs from having an enlarged prostate (subclinical prostatic inflammation) or may even result from prostatitis or a urinary tract infection [5]. Low scores (2-4) usually hint toward a probability of 12-23% of having prostate cancer, high scores (4-10) mean of having a probability of 25% of having cancer and lastly 10+ scores are considered to have greater than 50% probability of having prostate cancer [6]. In general, it is believed that the higher the PSA level test score is, the more likely the man is to have prostate cancer.

There are several ways to treat early stages of prostate cancer. The first is by radical prostatectomy, which is the removal of the cancerous prostate tissue or the entire prostate gland along with attached seminal vesicles and nearby lymph nodes. Another approach would be by radiation therapy which uses high powered rays to kill the cancerous cells [7]. There are also chemical means to treat prostate cancer- by hormone therapy or by chemotherapy, in which the patients are given anti-cancer drugs to kill the cancer cells, reduce the cancer, or relieve the symptoms of the cancer [8]. Prostate cancer grows when in contact with the male hormone testosterone and other androgens [9]. Hormone therapy adds, reduces or stops related hormones required for prostate cancer; this is usually done by giving the patient drugs to reduce the testosterone levels [10]. Chemotherapy is more often used to reduce metastasis or when hormone therapy is not effective. The patient is usually given drugs orally or intravenously – commonly used drugs are Docetaxel, Cabazitaxel, Sipuleucel, and Abiraterone [11].

Despite these very effective treatments, there are problems with failures of treatment and sometimes early detection. Some patients don't respond to available treatments and the level of resistance to chemotherapy varies in patients. Certain drugs may not be activated *in vivo* for effectiveness, may be inactivated by proteins produced in the body or may be degraded because of drug-metabolizing enzymes and, importantly, some tumors or a subset of tumor cells may be resistant to the drugs. While chemotherapeutic drugs may work initially, over time, the tumors may develop cross-resistance. An approach to overcome this resistance has been to use combinatorial drug therapy. However, using a high dose of chemotherapeutic agents is not only toxic to tumor cells but also surrounding normal body tissues and organs. Thus, it is urgent to find a non-toxic drug combination that targets only tumor cells [11]. Another approach to circumvent resistance is to identify genes that are responsible for the resistance, for example, by doing a differential analysis of gene expression in both drug-resistant and drug-sensitive tumor cells [13].

Our recent findings have identified one transcription factor, namely, Yin Yang 1 (YY1), that is overexpressed in prostate cancer and YY1 was found to regulate tumor cell growth and drug resistance[14,15]. A few reviews on YY1 have been published that cover some of the roles of YY1 in normal and cancer [16-18]. In this brief review, we have added additional information that has not been addressed previously. We will present studies on the role of YY1 in the pathogenesis of prostate cancer, its role in resistance, and findings on YY1 gene analysis in bioinformatics datasets.

### Yin Yang I (YY1)

#### A. General properties

YY1 is a transcriptional protein found in normal and cancerous cells and functions in opposing ways—as an activator and as a repressor [19] [20]. It has diverse biological functions and has a pleiotropic effect on promoters, and thus, gene expressions [21]. These effects make it difficult to assess whether the overexpression or the reduced expression of YY1 leads to or hinders oncogenesis. The YY1 structure is shaped in a very specific way. It has four zinc fingers, a protein motif that uses zinc ions to stabilize its structure that are attached to its carboxylic terminus. It has regions rich in alanine and glycine with 11 consecutive acidic residues and 11 consecutive histidine residues. The two zones are separated by a region loaded with glycine amino acids [17] [22]. Depending on the DNA-binding sites, the YY1 transcriptional protein can act either as an activator or as a repressor or even as an initiator protein. Most of the sequence that allows it to regulate as an activator is mapped near the 16-29 and 80-100 amino acid sequences [17].

Reported studies investigated the role of YY1 in metastasis, its role as a therapeutic target, and finally its significance and function as a diagnostic or a prognostic factor [14,16]. Briefly, we report various genes that are regulated by YY1. Such genes are involved in cytokinesis, apoptosis, cell development, cell differentiation, genes important as housekeeping genes and genes that regulate the cell cycle [23]. Several studies have been performed to delineate the function of the *YY1* gene within cells. Shi *et al* showed that YY1 was crucial for embryonic development. They used a homozygous mutation in mice which led to lethality and the heterozygous knockout suffered from growth retardation [23]. Heterozygous mutants showed transformation of their axial skeleton. Reducing the levels of YY1 impaired growth and its deletion lead to cytokinesis failure. Even though there is a strong correlation between YY1 and cell proliferation, the effect of dosage is still unclear [23].

Noteworthy, recent interest has shifted due to YY1 level of expression and its various functions in different cancers. For example, YY1 has been shown to regulate many genes that are implicated in cancers such as *c-myc, c-fos, EIA, p53, ERB B2* [15,22]. In addition, YY1 regulates several genes, not through transcriptional regulation, but as a cofactor interacting with other proteins that regulate cell proliferation and apoptosis. Among these genes are *p53, Mdm2, Ezh2, R6, caspases and HDACs* [15,24].

Most reported studies showed that YY1 acts as a transcription factor, an activator or a repressor, through directly binding to promoters or as a cofactor [15,22]. For instance, Luke *et al* [25] reported that YY1 is required for the recruitment of HDAC2 by HoxA11 via enhancing the HoxA11-DNA binding. In addition, YY1 stimulates Mdm2-mediated p53 ubiquitination and degradation [15,22,26]. Studies by Lee *et al* [27] and Yang *et al* [28] reported the association of YY1 with p300 and HDACs resulting in histone acetylation and deacetylation, respectively.

YY1 has contradictory-mediated effects. One example is the interaction between the Hepatitis C virus core and YY1. YY1 recruits a coactivator, p300, and a nucleolar phosphoprotein, B23, to attach it to its binding site and activates gene expression when the HCV core is present. HCV interacts directly with the C-terminal end of B23 and uses it to relieve the repression effect of YY1 on B23. When the core is absent, YY1 recruits histone deacetylase 1(HDAC1) to repress the *B23* gene, a gene which suppresses multiple tumor suppressors and, hence, exhibits oncogenic effects. The repression of such a gene leads to a stronger role of YY1 in liver tumorigenesis [18]. DeNigris *et al* have reported that, in general, overexpression of the YY1 protein has a tendency to serve as an activator of tumorigenesis and that it is associated with malignancy of bone tumors [29]. Deletion of YY1 reduces cell invasion and metastatic growth significantly [24]. In this case, YY1 repressed the tumor suppressing gene and increased the expression of oncogenes. There are also cases in which YY1 expression positively correlates with the expression of a tumor suppressor gene and hinders tumorigenesis. For example, Wang *et al* showed that HLJ-1 is a tumor suppressor that inhibits tumor angiogenesis and metastasis and that the overexpression of YY1 with another activator protein (AP)-1 causes the initiation of the transcription for HLJ-1 and enhances the transcription by a 3-fold increase [18,30].

Metastasis is the process during which primary tumors break off and invade normal tissues at distant sites leading to the production of secondary tumors. It is important to figure out the location and identification of primary tumors before they attack other locations since most patients are diagnosed with cancer at a time after the cancer had spread [31]. It has become even more important not to just find a therapy that targets primary tumors but also target pathways that lead to metastasis. Bonavida and Baritaki [31] have investigated the role of YY1 in the initiation and development of cancer metastasis. They have found that the YY1 factor participates in a metastatic cascade and regulates the epithelial to mesenchymal transition (EMT) and corroborated by the use of various inhibitors and regulators in tumor cell lines [31]. Snail, a metastasis inducer, represses the suppressor gene product, Raf-kinase inhibitor protein (RKIP) that inhibits both Raf-1/MEK/ERK and NF-κB survival pathways used in EMT [32]. This impairs the circuitry that involves the NF-κB/Snail/RKIP loop [31] and EMT and maintains the chemotherapeutic drug resistance [33].NF-κB regulates the transcription of YY1 and YY1 regulates the transcription of Snail and Snail represses RKIP [34]. Therefore, if NF-κB is inhibited, it will also lead to the inhibition of Snail and YY1 and the induction of RKIP which will lead to the inhibition of EMT. It is this dysregulated EMT circuitry that is commonly found in tumor cells [17,35].

#### B. Significance of YY1 expression in various cancers

#### 1. All cancers (excluding prostate cancer)

The expression and activities of YY1 are reported in both normal and cancerous cells. YY1 may activate one or more genes while deactivating another gene(s) that will result in a different phenotypic effect [36]. Most cancers show increased expression of YY1 while a few show a lowered expression. An overexpression of YY1 was related to exhibit a strong correlation in patients with osteosarcoma, an aggressive bone tumor that occurs when a teenager matures into an adult, and it does this by silencing target genes [37]. The increased expression of YY1 was reported in prostate cancer [38-41], colon cancer [39,42], ovary cancer [39,40], breast cancer [42,43], skin cancer [40,43], cervix cancer [40,44], bladder cancer [39,40], bone cancer [45], liver cancer [39], and lung cancer [39]. In contrast, a reduced expression of YY1 has been reported in melanomas, urothelial carcinomas and osteosarcomas [17].

Wu *et al* showed that in hypoxic stress conditions in lung cancer, the inhibition of YY1 reduced the buildup of an important transcription factor, HIF-1α, in a p53-independent manner and that the inhibition of YY1 also resulted in the suppression of the metastatic potential in cancer cells [46]. HIF-1α has been known to be closely associated with tumor growth and metastasis and the reduction of HIF-1α would directly lead to reduced tumorigenesis and decrease of metastasis [47]. To study this relationship, Wu *et al* [46] investigated the effect of YY1 silencing under hypoxia by making shRNA vectors to suppress the expression of YY1. Cell lines were then transfected with these vectors and after 48 hours of hypoxia, the proliferation rate of these cell lines was examined. The results demonstrated that in a long period of hypoxic condition, YY1 silenced cell lines suppressed the colonization and proliferation of cancer cells in the lungs. When the same experiment was done after 6-24 hours of hypoxia, there was no significant change in the proliferation rate of cancer cells. These results, nonetheless, indicate the importance of YY1 expression in the growth of lung cancer [46].

The human epidermal growth factor receptor 2 (HER2) is a protein that is commonly overexpressed in high grade breast cancer cells and is frequently associated with tumor growth and metastasis [48,49]. The AP-2 family contains a group of proteins- four of which have been identified in the promoter of the *HER2* gene and YY1 has been shown to play a critical role as a cofactor in stimulating AP-2 transcriptional activity. Powe *et al* studied the implications of using YY1 and AP-2 as therapeutic targets in breast cancer and found that AP-2 alpha/beta correlated significantly with YY1 and could be used as important markers for the prognosis of cancer [50].

Human meningiomas and astrocytic gliomas are the most common type of cancers found in the central nervous system (CNS) and make up 20% and 35% of all CNS tumor cases, respectively [51,52]. Most meningiomas have been shown to be benign but quickly grow into large anaplastic meningiomas that are dedifferentiated and increasingly multiplying tumors [53]. Baritaki *et al* [54] quantified the expression of YY1 in tissues from low grade gliomas, bening meningiomas, and glioblastomas multiforme (GBMs) using real time polymerase chain reaction. They observed a positive correlation between the expression of YY1 and the progression of gliomas and meningiomas and concluded that YY1 would serve as a good potential therapeutic target against brain tumors. This supports the observations studied in recent studies illustrating elevated YY1 expression levels in various tumors and carcinomas. These investigators suggested that these expression levels stay elevated during the progression of these gliomas and meningiomas and serve as a sign of disease development [54].

YY1 overexpression in ovarian cancer, in contrast to most cancers, correlated with higher ovarian cancer survival rates [55]. Matsumura *et al* [56] studied YY1 function using siRNA knockdowns and the microarray analysis showed a strong positive correlation between binding motifs in ovarian cancer lines and YY1 expression. High YY1 correlated to a high E2F3 activity which led to a higher rate of survival among patients that were given a chemotherapeutic treatment- specifically paclitaxel [56]. Unlike in the cancers discussed above in which high YY1 expression indicates increased cell proliferation and metastasis, in ovarian cancer, however, high YY1 expression correlates with the inhibition of proliferation and motility and higher sensitivities to taxanes, a group of drugs that is widely used as chemotherapy agents. Although it is presently difficult to differentiate what causes this association between high YY1 activity and taxane sensitivity, they suggested that it may be due to the fact that taxanes specifically target microtubules [57]. Because YY1 suppression inhibits cancer cell migration and that the migration is strongly associated with microtubule dynamics, YY1 may have a role in the transcription of proteins that are involved in microtubule dynamics and, hence, also in metastasis of the ovarian cancer cells [56,58]. Matsumura *et al* also found that there is an association between YY1 and E2F, a transcription factor that is critical in the control of the cell cycle and tumor suppressing protein actions [56].

#### 2. Prostate cancer

Having a certain level of YY1 expression is considered normal within certain cells and is necessary for the YY1 to maintain its function. Deng *et al* [24] studied the interaction between the androgen receptor (AR) and YY1 in prostate and observed, through *in vitro* protein studies and immunoprecipitation, that there is in fact a protein-protein interaction between the AR and YY1. The AR regulates prostate development and its interaction with YY1 optimizes its transcriptional activity. They observed that a normal amount of YY1 within prostate cells does in fact optimize its transcriptional activity but overexpression of YY1 causes it to lose its function. When YY1 is overexpressed, the excessive YY1 interacts with the AR and other cofactors which then interfere with the formation of a transcription complex. Consequently, not only does this keep the complex from optimal transcriptional activity but causes an inverse effect on the expression of AR-targeted genes due to the squelching effect of excessive amount of YY1 present [24,59].

#### a. YY1 vs PSA [62]

Androgens interaction with the AR on prostate cancer cells stimulate proliferation and progression [60]. The transcription factor AR is a nuclear receptor and contains two activated domains (AF1 and AF2), a DNA-binding domain and a ligand-binding domain [61]. The binding of androgen to AR changes its conformation and composition of the AR-containing complex and resulting in its translocation from the cytoplasm into the nucleus. Nuclear AR associates with androgen responsive elements (AREs) in the promoter and enhancer regions of its target genes and stimulate their expression. Denz *et al* [62] studied the relationship between YY1 and AR in prostate cancer. Several findings were observed, namely, (1) YY1 directly interacts physically with AR (2) YY1 enhances the transcriptional activity of AR (3) knockout of YY1 decreases the expression of PSA (4) the enhancement of AR by YY1 is not due to YY1 DNA-binding but to the YY1-AR complex and (5) the complex of YY1 with AR is required for the association of AR with AREs. Overall, this study demonstrated that YY1 is pivotal for AR-mediated transcriptional activity. Thus, the YY1-AR complex is not YY1-DNA associated and is responsible for the activation of PSA.

#### b. YY1 vs PSCA [68]

PSCA is a GP1-anchored cell surface protein [63] and is a marker of the transiently amplifying cell population within the prostate epithelium [64]. Overexpression of PSCA is observed in a subset of prostate cancer of all stages from PIN to metastatic disease [65]. The function of PSCA is still unknown. The expression of PSCA in PIN is a predictor of late development of invasive adenocarcinoma [66]. *PSCA* is an androgen-responsive gene through the interaction of AR with AREs [67]. Other regulations might be involved since the PSCA is expressed in castration-resistant prostate cancer. YY1 is overexpressed in PIN and prostate cancer [62]. YY1 also regulates PSA via its interaction with AR. Its role in regulating PSCA was studied by Tang *et al* [68] who reported that (1) recombinant YY1 binds to binding elements in the human PSCA promoter (2) *in vivo*, YY1 interacts with the PSCA protein as assessed by chromatin immuno-precipitation assay (3) much of YY1 binding sites demonstrated that YY1 has a dual opposing control of PSCA activation; activation of both binding sites generated intermediate luciferase activity (4) YY1 knockdown induces androgen-mediated upregulation of PSCA and (5) YY1 siRNA in PC3 (AR-mutated) increased endogenous PSCA expression.

#### c. Role of YY1expression in prostate cancer [18,69]

Seligson *et al* [14] were the first to report on the overexpression of YY1 in Welsh cancer compared to normal tissues. The protein level of YY1 was a predictor of recurrent disease and a low level of nuclear staining was associated with a shorter time of recurrence. Ren *et al* [20] investigated the role of YY1 and HOXB 13, a homeo-domain protein thought to play a role in growth arrest in AR negative prostate cancer cells. Co-immunoprecipitation assays demonstrated the presence of an HDAC4/ YY1 complex and HDAC4 represses HOXB13. YY1 facilitated the recruitment of HDAC4 to HOXB13. The HOXB13 promoter has two YY1-binding sites essential for recruitment of YY1 and HDAC4. This finding demonstrated that YY1 and HDAC4 promote growth of AR negative prostate cancer cells by repressing the transcription of regulation of HOXB13 [20].

Sun *et al* [70] carried out a protein analysis comparing the benign prostatic hyperplasia and prostate cancer. There were 20 proteins upregulated and 26 downregulated between benign and prostate cancer. Gene ontology analysis revealed that activation of SP1, p53, YY1, AR, and c-Myc were involved in the initiation of the five transcriptionally regulated networks [70]. These findings suggested that YY1 is a pivotal factor in the development of prostate cancer.

### Regulation of Tumor Resistance in Prostate Cancer by YY1

Treatment of various cancers includes surgery, chemotherapy, radiation, immunotherapy and combinations thereof. These various modalities result in significant clinical responses and seldom cures. However, an initial subset of cancer patients remains unresponsive and another responding subset develops resistance and often cross-resistance to different therapeutic modalities. Clearly, these subsets of patients require novel treatments that can overcome resistance. Approaches that are currently being investigated consist of targeting resistance factors directly at the tumor cells or indirectly at the tumor microenvironment. Targeting resistance factors include agents that interfere with constitutively activated survival/anti-apoptotic pathways that usually control resistance. Our laboratory has examined a gene product, namely, YY1, that is overexpressed in many cancers including prostate cancer. We have reported that YY1 is involved in the regulation of both drug and immune resistance.

### A. Role of YY1 in the regulation of immune resistance

We have reported that tumor cells that are resistant to FasL-mediated apoptosis can be sensitized following treatment with interferon-gamma (IFN-γ). One of the many downstream targets of IFN-γ is the enzyme iNOS and, therefore, we examined the role of iNOS in the regulation of tumor cell resistance to FasL. The findings demonstrated that the induction of iNOS following treatment of tumor cells with IFN-γ or treatment with NO donors sensitized the cells to FasL apoptosis. The sensitization was correlated with upregulation of Fas on the cell surface membrane of treated tumor cells. Analysis of the Fas promoter revealed many putative transcription factor-binding sites including for YY1. We established the role of YY1 in the resistance by demonstrating that it is inhibited by IFN-γ, iNOS and NO donors and, also, YY1 represses the transcription of Fas. In addition, treatment of tumor cells with siRNA YY1 resulted in the up regulation of Fas and sensitization to FasL apoptosis [71]. These findings demonstrated the pivotal role of YY1 in the regulation of resistance to FasL apoptosis. Subsequent studies demonstrated the resistance to tumor cells to TRAIL apoptosis. We have reported that TRAIL apoptosis was regulated, in part, by YY1. The inhibition of YY1 sensitized the tumor cells to TRAIL apoptosis. We also reported that YY1 represses the TRAIL receptor DR5 through its binding to the DR5 promoter [35,71]. The sensitization to TRAIL apoptosis correlated with the upregulation of DR5. Overall, the above findings established the direct role of YY1 in the regulation of tumor cells resistance to FasL and TRAIL apoptosis.

### B. Role of YY1 in the regulation of chemoresistance

Wink *et al* [72] demonstrated that NO can sensitize tumor cells to various chemotherapeutic agents when used at high concentrations on human prostate carcinoma lines. We have reported that treatment of tumor cells with the NO donor, DETANONOate, resulted in the S-nitrosylation of several proteins including YY1 [73]. The nitrosylation of YY1 resulted in the loss of its transcriptional activity, a mechanism by which the tumor cells exhibit the repression of both Fas and DR5 receptors. Further, NO nitrosylates NF-κB (p50 and p65) and results in its loss of activity and inhibition downstream of several target genes involved in survival and anti-apoptotic activities [74]. Our studies corroborated the findings above by Wink *et al*. and demonstrated that treatment of DU145 human prostate carcinoma cells with the NO donor DETANONOate sensitized the drug resistant tumor cells to apoptosis to various chemotherapeutic agents [75]. The sensitization of tumor cells to CDDP by DETANONOate was the result, in part, in the inhibition of NF-κB, YY1 and anti-apoptotic gene products such as Bcl-2 and Bcl-xl. The *in vitro* findings were corroborated *in vivo* in mice bearing a PC3 tumor xenograft and treated by the combination of DETANONOate and CDDP [76].

Altogether, the above findings demonstrated clearly that YY1 plays a direct role in the regulation of tumor cells resistance to both chemotherapeutic drugs and immune-mediated apoptosis. These findings suggest the potential therapeutic application of YY1 inhibition in the reversal of patient tumors refractory to conventional treatments.

### Role of YY1 in the Regulation of Prostate Cancer EMT and Metastasis

Many solid cancers, in general, develop molecular, biochemical, genetic and epigenetic mechanisms to evade the response to the host immune surveillance and external cytotoxic activities [36]. These features also facilitate tumor spread from their primary site and activation of the epithelial to mesenchymal transition (EMT), and establishment of distant metastases. Several reports demonstrated the involvement of both tumor metastasis-inducing and metastasis-suppressor gene products that regulate the EMT and metastatic spread [77]. The EMT process consists of the conversion of the epithelial phenotype to the mesenchymal phenotype through complex gene regulatory mechanisms. The EMT phenotype consists, in part, in the loss of epithelial markers such as E-cadherin and the gain of mesenchymal markers such as vimentin and fibronectin along with invasive properties. The hyperactivated constitutive NF-κB pathway in many cancers has been reported to be involved in the EMT switch through the regulation and expression of target genes such as the transcription factor Snail, which represses E-cadherin transcription [78]. Studies by us and others have examined the role of YY1 in the regulation of EMT in human prostate cancer cell lines. We established the dysregulation of the NF-κB/Snail/YY1/RKIP loop in cancer cells and demonstrated its role in the regulation of EMT. Briefly, NF-κB regulates both Snail and YY1; YY1 regulates Snail; and Snail regulates RKIP, a metastasis suppressor gene product that in turn inhibits both the NF-κB and the Ras/MEK pathways. Hence, this dysregulated loop promotes EMT and each gene product in the loop alone is also involved in the regulation of EMT [31]. The role of YY1 in the regulation of EMT was established by demonstrating that its inhibition reverses EMT to MET [31]. Thus, our findings with YY1 demonstrate that YY1 exerts pleiotropic activities and that it regulates immune resistance, drug resistance and EMT. Accordingly, targeting YY1 may result in a double sword effect, namely, reversal of resistance and reversal of EMT.

### Analysis of YY1 Gene Expression in Prostate Cancer by Bioinformatics

A study of YY1 expression levels in various carcinomas in the Oncomine Bioinformatics Database reaffirms the predicament researcher's face in giving YY1 a good or bad label. While high YY1 expressions in lung, breast, and brain and CNS cancers correspond to a high likelihood of cancer metastasis, high expressions of the same YY1 in ovarian cancer increases survival rates [56].

Oncomine Bioinformatics Database is a cancer microarray database that is useful to perform differential analyses of gene expression using data from hundreds to thousands of patients. It permits researchers to get a comprehensive idea of gene expression by searching the database for specific mRNA levels of genes in normal tissues versus cancerous tissues in various kinds of cancers. Researchers upload their data from their experiments after their publication for the general public. Of importance, researchers could use these data to their full potential and can demonstrate trends in gene expression levels in different cancer types and use the data to look for diagnostic or prognostic markers and potential therapeutic targets since all of the data are available with publication references, supplementary data, and annotations [79].

Because the above data are uploaded at different times and the samples used have been exposed to different experimental methods, the data are expected not to be uniform and may be dissimilar. What is clear is that each dataset may have been interpreted by the investigators in a certain way but clearly many more alternative interpretations may derive when all the datasets are examined concurrently.

**Figure 1:**
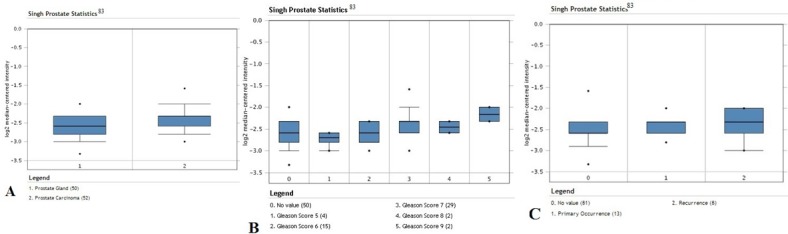
Overexpression of YY1 in prostate cancer in datasets by Singh *et al* [83]. Analysis of data for prostate cancer; 102 surgical specimens from radical prostatectomy were used for data analysis. A. Expression levels of the YY1 transcription factor in prostate gland normal tissues (n =50) versus prostate carcinoma tissues (n =52). Significant increased expression in carcinoma tissues compared to normal tissues (p-value = 4.51 × 10^−5^). B. Expression levels of YY1 according to Gleason score (Gleason scores 5-9). C. Expression levels of YY1 in primary (n = 13) and recurrent tumor tissues (n = 8). There seems to be no apparent change in the average expression level.

**Figure 2:**
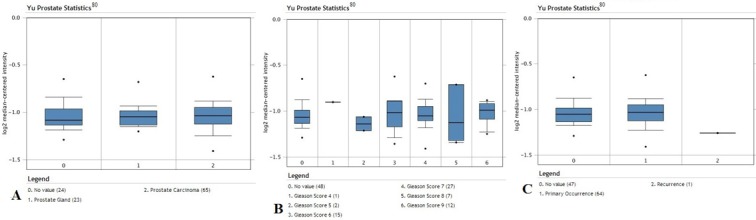
Overexpression of YY1 in prostate cancer in datasets by Yu *et al* [80]. Analysis of data for prostate cancer; 112 surgical specimens from radical prostatectomy were used for data analysis. A. Expression levels of the YY1 transcription factor in prostate gland normal tissues (n =23) versus prostate carcinoma tissues (n =65). There is no significant change in the average expression levels between normal tissues and carcinoma tissues (p-value = 0.022). B. Expression levels of YY1 according to Gleason score (Gleason scores 4-9). There seems to be oscillation in the expression values at various Gleason scores. C. Expression levels of YY1 in primary (n = 64) and recurrent tumor tissues (n = 1).

**Figure 3:**
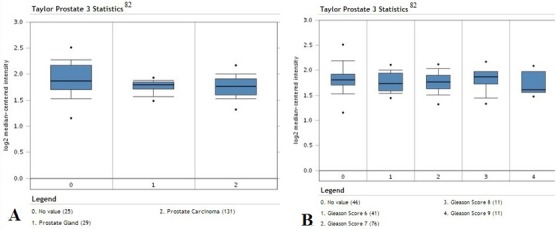
Similar YY1 expression in prostate cancer in datasets by Taylor *et al* [82]. Analysis of data for prostate cancer; 179 surgical specimens from radical prostatectomy and 6 cell lines used for data analysis. A. Expression levels of the YY1 transcription factor in prostate gland normal tissues (n =29) versus prostate adenocarcinoma tissues (n =131). There is no significant change on the expression levels between normal tissues and adenocarcinoma tissues. B. Expression levels of YY1 according to Gleason score (Gleason scores 6-9).

**Figure 4:**
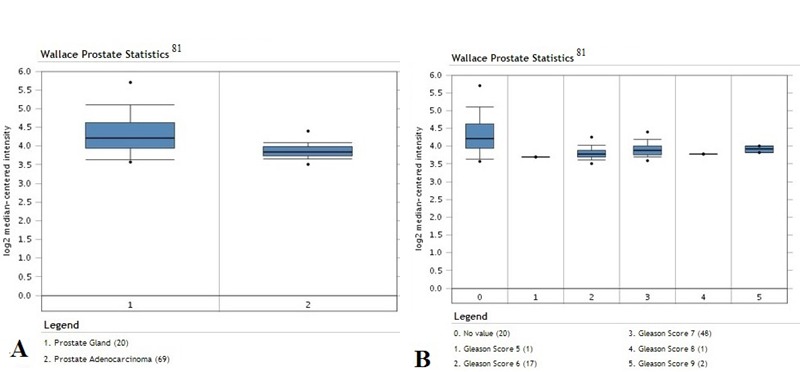
Similar YY1 expression in normal prostate cancer in datasets by Wallace *et al* [81]. Analysis of data for prostate cancer; (surgical specimens from radical prostatectomy, 2 pooled biopsy specimens, and 18 tissue specimens used for data analysis). A. Expression levels of YY1 in prostate gland normal tissues (n =20) versus prostate adenocarcinoma tissues (n =69). Significant reduced expression in carcinoma tissues compared to normal tissues (p-value = 7.41 × 10^−4^). B. Expression levels of YY1 according to Gleason score (Gleason scores 5-9).

We have analyzed the YY1 mRNA expression levels using available public datasets of microarrays that were retrieved from the Oncomine Database and gene expression Omnibos, derived from the published analyses reported by several investigators [80-83]. The findings are briefly summarized in Tables 1 and 2. Clearly, the analyses demonstrated the lack of uniformity and concordance of the expression levels of YY1 in prostate cancer by different investigators. Some datasets demonstrated overexpression of YY1, others demonstrated similar expression levels as normal prostate controls, and a few demonstrated decrease in YY1 in prostate cancer compared to normal prostate epithelium. It is not clear why these differences have been noted in various investigations. It may be assumed that there exist differences in tumor tissues obtained for microarray analyses and other factors of unknown origins. We found that 5 of the 14 datasets reported overexpression of YY1 in prostate cancer and a representative is shown in Figure 1. Seven datasets reported no differences and are represented in Figures 2 and 3. The underexpression of YY1 of two datasets are represented in Figure 4. (Table 1) We have also analyzed the expression levels of YY1 as a function of the Gleason scores and the findings are summarized in Table 2. Briefly, the findings demonstrate there was an increase level of YY1 with progression in the datasets reported by Singh *et al* [81] and Ambs *et al* [83].

**Table 1:** Lack of Concordance on YY1 Overexpression Levels in Prostate Cancer in Various Datasets (2001-2012)

Year	Oncomine Name	# of Carcinomas	Prostate Cancer vs. Normal Prostate
2001	Welsh Prostate [84]	25	Underexpression, p = 0.009
2002	Singh Prostate [83]	52	Overexpression, p = 0.00004
2002	Luo Prostate [85]	15	No change, p = 0.090 (NS*)
2002	LaTulippe Prostate [86]	23	No change, p = 0.141 (NS)
2003	Vanaja Prostate [87]	27	Overexpression, p = 0.004
2004	Yu Prostate [80]	65	No change, p = 0.022
2004	La Pointe Prostate [88]	54	No change, p = 0.163 (NS)
2005	Varambally Prostate [41]	7	No change, p = 0.176 (NS)
2006	Bandyopadhyay Prostate (Liu)[89]	44	No change, p = 0.098 (NS)
2007	Tomlins Prostate [90]	26	Overexpression, p = 0.016
2008	Ambs Prostate (Wallace)[81]	69	Underexpression, p = 0.0007
2009	Arredouani Prostate [91]	44	No change, p = 0.032
2010	Taylor Prostate [82]	131	No change, p = 0.578 (NS)
2012	Grasso Prostate [92]	59	No change, p = 0.612 (NS)

* NS = Not Significant

**Table 2:** YY1 Expression as a Function of Gleason Score in Various Datasets (2002-2012)

Year	Oncomine Name	Specimen	Gleason Score	YY1 Expression
2002	Singh Prostate [83]	Surgical Specimens (102)	(5-9)	Increasing
2004	Yu Prostate [80]	Surgical Specimens (112)	(4-9)	No change
2004	La Pointe Prostate [88]	Surgical Specimens (93)	(6-9)	No change
2006	Bandyopadhyay Prostate (Liu)[89]	Surgical Specimens (57)	(6-8)	No change
2008	Ambs Prostate (Wallace)[81]	Pooled Biopsies (2) Surgical Specimens (69) Tissue Specimens (18)	(5-9)	Increasing
2010	Taylor Prostate [82]	Cell Lines (6) Surgical Specimens (179)	(6-9)	No change
2012	Grasso Prostate [92]	Surgical Specimens (122)	(7,8) only 3 values in chart	Decrease

## CONCLUDING REMARKS

This review has briefly discussed the role of YY1 in the pathogenesis of prostate cancer as a model. Clearly, overexpression of YY1 has been noted in many cancers and was shown to play a pivotal role in the regulation of EMT and metastasis as well as its role in the regulation in tumor cell resistance to both chemotherapy and immunotherapy. Therefore, YY1 is proposed to be a therapeutic target for intervention as well as a potential prognostic biomarker in prostate cancer. There are several agents that inhibit YY1 expression such as proteasome inhibitors [54], nitric oxide donors (NO) and siRNA [76]. These agents will sensitize the tumor cells to cytotoxic drugs-induced apoptosis and inhibit EMT and metastasis. We suggest that researchers and scientists need to validate the findings observed *in vitro* by using preclinical animal models bearing human tumor xenografts. We also suggest that it is important to find out whether the NF-κB/Snail/YY1/RKIP/PTEN dysregulated circuitry found in the EMT phenotype is also shared in cancer stem cells such that YY1 inhibitors will be helpful to prevent the development of a metastatic tumor from stem cells and induce the death of stem cells [31]. We also believe that the findings reported in prostate cancer may be also found in many other cancers and, therefore, therapeutic inhibitors of YY1 may be of general use for many cancers.
